# Optimizing breastfeeding for hospitalized newborns: A narrative review of midwifery-led interventions

**DOI:** 10.18332/ejm/200341

**Published:** 2025-02-21

**Authors:** Kira Madeleine Harting, Dominique Singer, Julia Heiter

**Affiliations:** 1 Division of Obstetrics and Fetal Medicine, University Medical Center Eppendorf, Hamburg, Germany; 2 Division of Neonatology and Pediatric Intensive Care, University Medical Center Eppendorf, Hamburg, Germany

**Keywords:** midwife, breastfeeding success, breastfeeding support, hospitalized newborns, neonatal ward

## Abstract

Breastmilk is the best source of nutrition for newborns. The World Health Organization recommends exclusive breastfeeding for the first six months of life as it has benefits to mother and child. However, breastfeeding can be challenging. Especially when the newborn is hospitalized, the physical separation of mother and child can make breastfeeding difficult. Hence pre- and post-natal midwife care supporting breastfeeding become more important. The aim of this narrative review is to identify measures taken by midwives in the labor ward, on the postpartum unit and at home that can influence breastfeeding success positively in hospitalized newborns. A literature review was conducted in PubMed and CINAHL and on the website of the European Institute for Breastfeeding and Lactation from April to September 2023. Studies from 2013 to 2023 written in German or English comparing two different measures/groups were considered. Twenty studies were included and five measures, taken by midwives, were identified. Skin-to-skin contact leads to higher (exclusive) breastmilk feeding rates, better sucking behavior and a shorter time to full enteral feeding. Regular breastmilk expression and supervised breastfeeding attempts result in higher breast milk feeding rates. Breastfeeding counselling enables the mothers to access lactation education. Uninterrupted visiting hours lead to higher exclusive breastmilk feeding rates. Midwives play a key role in promoting breastfeeding among hospitalized newborns involving initiating lactation, strengthening the mother–child bond and providing appropriate breastfeeding advice. There is a further need for research, as midwives are rarely involved in studies.

## INTRODUCTION

The World Health Organization (WHO) recommends exclusive breastfeeding for six months. Breastfeeding should be continued with the introduction of complementary food up to the age of two and beyond, for as long as the mother and child wish^1^. A major obstacle to implementing these breastfeeding recommendations is the physical separation of mother and child when the newborn is admitted to the neonatal intensive care unit or neonatal ward directly after birth^2^.

Breastmilk has a protective effect for newborns^3^. Breastfed newborns are less likely to suffer from diarrhea and respiratory infections as well as otitis media in the first two years of life^4,5^. In the long-term, breastmilk feeding reduces the risk of being overweight or obese, and is associated with better cardiorespiratory fitness in children and adolescents^6,7^. In addition, newborns fed with mother’s own milk (MOM) are less likely to develop diabetes mellitus type II and chronic inflammatory bowel diseases^6,8^. The risk of leukemia is reduced when being breastfed for more than six months^9^. Breastfeeding for at least two months reduces the risk of sudden infant death syndrome (SIDS) by 50%^10^.

Premature infants should primarily be fed with breastmilk, as this influences their development into adulthood. If this is not possible, human donor milk (HDM) is an alternative. This is recommended not only by the WHO, but also by many other societies such as the American Academy of Pediatrics and the European Society for Pediatric Gastroenterology, Hepatology and Nutrition^11-13^. Being fed with MOM or HDM reduces the risk of necrotizing enterocolitis (NEC)^14,15^. Additionally, premature infants fed with MOM can receive complete enteral nutrition earlier^14^. Fewer cases of late-onset sepsis and retinopathies of prematurity have been found when the newborn is fed with MOM^14,16^.

For mothers, in the postpartum period, breastfeeding promotes uterine involution and reduces blood loss^17,18^. A longer duration of breastfeeding is associated with a lower risk of postpartum depression^19^. Breastfeeding also has long-term effects on maternal health such as a reduced risk of cardiovascular diseases, diabetes, endometrial, breast and ovarian cancer^20-22^.

The midwives’ care in Germany covers pregnancy, birth, the postpartum and breastfeeding period. They have a caring, advisory and observational role^23^. This continuous care has a positive effect on the duration of breastfeeding and breastfeeding satisfaction^24^. Mothers often experience short- and long-term breastfeeding problems due to the physical separation from their newborn when mother and child are admitted to different wards after birth^2^. There are no guidelines for midwives that describe the procedure for mothers who desire to breastfeed when the newborn is admitted to a neonatal ward. This review aims to identify evidence-based measures that are or can be carried out by midwives, which improve the breastfeeding success of hospitalized newborns, discussing the relevance of midwifery care in different settings.

A narrative review was chosen to give a comprehensive overview of this topic. It offers flexibility to include different study designs and insights from different disciplines. To narrow down literature, inclusion and exclusion criteria were defined as follows. In order to represent the current evidence, studies from 2013 to 2023 were considered. The studies included were written in German or English, and compared an intervention group with a control group or two different measures. Prospective studies were desirable, especially randomized controlled trials. Alternatively, meta-analyses were eligible. If no prospective studies were found, retrospective or cohort studies were included. Only studies that were fully accessible were included in this review. Each included study had a reference to breastfeeding success, which was recognized by the duration of breastfeeding, the amount of breastmilk obtained or the type of feeding the newborn at discharge.

A literature search was carried out in the electronic databases PubMed and CINAHL and the snowball method was used on the website of the European Institute for Breastfeeding and Lactation from April to September 2023. Key search terms used were ‘breastfeeding success’, ‘measures’ and ‘hospitalized newborns’. They were combined with the Boolean operators AND and OR (Supplementary file Table 1B).

The included studies were selected by one person; 4200 studies were identified out of which 2640 were published between 2013 to 2023. After evaluation of title and abstract, 55 studies seemed suitable and were evaluated in full text. To make the literature search more comprehensible a PRISMA flowchart (Supplementary file Figure 1) and search protocol (Supplementary file Table 1A) are provided. The results were grouped thematically.

The literature research identified 20 different studies, which can be divided into five main evidence-based measures that can be carried out by midwives to improve breastfeeding success in hospitalized newborns. Skin-toskin contact leads to higher (exclusive) breastmilk feeding rates, better sucking behavior and a shorter time to full enteral feeding^25-28^. Breast milk expression by collecting colostrum and by regular breastmilk pumping, as well as supervised first breastfeeding attempts result in higher breast milk feeding rates^29-39^. Breastfeeding counselling enables the mothers for better access to lactation education and more effective breastmilk collection^37,40-43^. Uninterrupted visiting hours result in higher exclusive breastmilk feeding rates^44^.

## OPTIMIZING BREASTFEEDING FOR HOSPITALIZED NEWBORNS

### Skin-to-skin contact

In a randomized controlled trial from India, published in 2014, the effects of early skin-to-skin contact on breastfeeding success in healthy, mature newborns were investigated. The intervention group (122 newborns) received skin-to-skin contact with the mother for at least two hours within 30 minutes of birth and was compared to the control group (118 newborns), who was measured immediately after birth, weighed, dressed, and then placed next to the mother. The newborns in the intervention group showed better sucking behavior at the breast in the first 24 hours (IBFAT mean score 9.55 vs 6.77; p<0.0001). After four to five days postpartum, more newborns in the intervention group (86.1%) were exclusively breastfed compared to the control group (66.9%) (p=0.002). At six weeks postpartum, a higher breastfeeding rate was found in the intervention group (85.2% vs 63.6%; p<0.0001). The authors of this study recommended the direct implementation of skin-to-skin contact^25^.

In 2022, a non-randomized, controlled study from China with a total of 182 participating mothers and their newborns showed similar results. The study examined the effect of direct postpartum skin-to-skin contact after birth for at least 90 minutes and the timing of the first breastfeeding session on breastfeeding success at discharge from hospital. In comparison, the newborns in the control group were measured, weighed and kept warm on a heated table and only had skin-to-skin contact with the mother after 90 to 120 minutes. A significant difference was found in the early initiation of the first breastfeeding session (p<0.001); 75.8% of the intervention group tried to breastfeed within the first two hours for the first time compared to 42.9% in the control group. The first breastfeeding was more successful in the intervention group (91.2% vs 74.7%; p=0.003). Exclusive breastfeeding at hospital discharge was observed more frequently in the intervention group (73.6% vs 44.0%; p<0.001)^26^.

In a randomized controlled study conducted in Egypt and published in 2020, the effect of skin-to-skin contact of preterm infants between 31 and 35 weeks of pregnancy on breastfeeding success was examined; 120 premature babies included in the study were divided into three groups. Group one received skin-to-skin contact for 60 minutes per day for at least 7 days, group two for 120 minutes per day for at least 7 days, and group three was in the parents’ arms for a maximum of 15 to 30 minutes per day. The preterm infants with skin-to-skin contact for 60 and 120 minutes showed better sucking behavior and a shorter time to complete enteral feeding than those who were only in their parents’ arms (IBFAT mean score: 60 min, 9; 120 min, 10.7; control group, 6.6; p<0.001)^27^.

Another study from India published in 2017 also showed an increase in exclusive breastfeeding rate in premature babies who received skin-to-skin contact. The randomized controlled trial with 160 newborns with a birth weight between 1000 g and 1800 g, compared the effect of early skin-to-skin contact for at least four hours a day within the first four days of life to late skin-to-skin contact on exclusive breastfeeding. In this study, late skin-to-skin contact meant that the premature infants only had skin-to-skin contact after complete stabilization (no more ventilation and no more infusions). Significant differences were found. In the early skin-to-skin contact group 86% were fed with breastmilk during hospitalization and 49% were breastfed, whereas in the late skin-to-skin contact group, 45% were fed exclusively with breastmilk and 30% were breastfed (exclusive breastmilk feeding, p<0.001; breastfeeding, p=0.021). Furthermore, more newborns in the early skinto-skin contact group were mainly fed with breastmilk one month after discharge (73% vs 36%; p<0.001)^28^.

### Breastmilk expression

A prospective cohort study was conducted in Sweden in 2015 with 138 preterm infants born before 32 weeks of gestation. Preterm infants, who received MOM on the third and seventh day postnatal where more likely to be exclusively breastmilk fed at a corrected age of 36 weeks’ gestational age (OR=1.18 per 10 mL/kg MOM; 95% CI: 1.06–1.32; p<0.001). This demonstrates the need for early initiation of breastmilk expression^29^.

In an observational study from the US, published in 2012, 67 participating mothers were asked to pump breastmilk every 8 hours for 15 minutes per day using a double pump set and to express breastmilk manually as often as possible in the first three days postpartum. This study showed that a combination of regular pumping and additional frequent hand-expression in the first few days is important for long-term breastfeeding success. Mothers who hand expressed breastmilk more than 5 times a day were able to express significantly more breastmilk in the first 8 weeks than mothers who hand expressed less than twice a day or between 2 and 5 times a day (mean milk production per day hand expression <2 times per day, 658 mL per day; hand expression >5 times per day 955 mL per day)^30^.

### Colostrum collection prepartum

Newborns to diabetic mothers often need colostrum at an early stage. A multi-center, unblinded, randomized controlled trial from Australia in 2017, examined the safety and efficacy of prepartum colostrum collection after 36 weeks of gestational age for mothers and their newborns; 635 pregnant women participated and were divided into two groups. The study showed that if the mother wishes to breastfeed, it is desirable for women with gestational diabetes and a low-risk pregnancy to collect colostrum after 36 weeks of pregnancy. There is no significant difference in newborns admitted to NICU nor between the two groups (RR=1.06, 95% CI: 0.66–1.46) but prepartum colostrum collection can prevent postpartum formula supplementation for the newborns^31^.

Whether prepartum colostrum collection is generally useful was investigated in a randomized, controlled pilot study conducted in the US in 2022 with 45 pregnant women. The intervention group was asked to perform breast massage and manual colostrum collection once or twice a day between the 37th and 40th week of pregnancy. This showed no risk for pregnant women with or without diabetes^32^.

### Expressing breastmilk by pumping

In a randomized controlled trial from the US in 2015, exclusive hand expression versus exclusive pumping was investigated in the first seven days. In both groups, the intervention took place every 3 hours for 15 to 30 minutes. Mothers who pumped breastmilk in the first 7 days were able to express significantly more breastmilk within the first 7 days (p<0.05) and higher amounts of breastmilk were observed after 28 days^33^.

Another observational study examined the timing of breastmilk collection in relation to the start of lactogenesis II in very low birth weight newborns. The 40 participating mothers were asked to express breastmilk at least 8 times a day for 15 minutes, using a double pump set. Mothers who had started obtaining breastmilk within the first hour collected more than twice as much breastmilk on day six and seven compared to the second group (294.0 mL and 306.2 mL vs 87.1 mL and 125.7 mL) (day six, p=0.003; day seven, p=0.005). Even after three and six weeks, significantly larger quantities of breastmilk were observed in mothers who had started collection within the first hour (543.5 mL and 440.0 mL vs 224.3 mL and 258.7 mL) (week three, p=0.007; week six, p=0.024). In addition, more mothers in the first group were still lactating after three and six weeks^34^.

An observational study conducted in Australia in 2019 assessed the effects of pumping management; 25 mothers of preterm infants were observed and the pumping frequency, pumping intervals and breastmilk volumes on day 10 and days 15 to 20 were documented. The study showed that mothers should pump breastmilk at least 5 times a day and take a maximum break of 7 hours between pumping to produce more breastmilk per day. Breastmilk expression 4 to 5 times a day makes a significant increase in daily breast milk volume (95% CI: 42–298, p=0.002) whereas breastmilk expression between five to six, seven, eight or nine times a day makes no significant difference (95% CI: -317–332, p=1)^35^.

The use of a single or double pump influenced the amount of breastmilk. This was investigated in a British randomized trial in 2016 with 62 participating mothers in two hospitals who had given birth before the 34th week of pregnancy. Significantly larger quantities of breastmilk were found within the first 10 days postpartum and when the newborn was discharged from the neonatal intensive care unit, when a double pump was used (p=0.003; 95 % CI: 80–356). The median was 288 mL (double pump) versus 147 mL (single pump) within the first 10 days, and 561 mL (double pump) versus 220 mL (single pump) at discharge^36^.

### First breastfeeding attempts

Direct breastfeeding during hospitalization compared to breastmilk feeding was associated with higher breastmilk feeding rates at discharge in a retrospective study from the US in 2022 with 865 newborns^37^. Another retrospective study from the US published in 2017 with 162 participating mothers who had given birth before the 32nd week of pregnancy, came to similar results. The mothers who fed their children with breastmilk for longer than 6 months were more likely to have breastfed their children during their stay in the neonatal intensive care unit (p=0.001; OR=5.54; 95% CI: 2.00–15.37)^38^. A randomized study of 33 preterm infants less than 34 weeks gestational age published in Canada in 2021, examined the effects of non-nutritive sucking on the empty breast versus pacifier on exclusive breastfeeding at discharge. Non-nutritive sucking was performed for 15 minutes a day until the infants were allowed to suck on the full breast. Upon discharge from hospital, more infants who had sucked on the empty breast were exclusively breastfed (63% vs 24%; p=0.037)^39^.

### Breastfeeding counselling

In a retrospective study conducted in the US in 2022, staff were questioned six months before and six months after the introduction of breastfeeding counselling on their own and parents’ access to breastfeeding knowledge and the efficiency of breastmilk collection. Additionally, breastfeeding consultation, access to breastfeeding knowledge and the provision of breastmilk were examined in a total of 161 newborns who were admitted to hospital. Access to lactation education increased significantly from 17% to 70% (p<0.0001), so the staff were more confident to give breastfeeding consultations. Moreover, effective breastmilk collection within the first 12 hours increased from 46% to 61% (p<0.01)^40^.

A retrospective study conducted in the US in 2019 of 167 hospitalized newborns (hospital A with 48 newborns and hospital B with 119 newborns) investigated the effect of breastfeeding counselling on breastfeeding success and breastmilk production in two different hospitals. Hospital A had lactation consultants working directly in the NICU and Hospital B had lactation consultants working only in the postpartum unit. It was found that the support of lactation consultants directly in the NICU significantly increased the rate of newborns/infants who were breastfed at discharge (p=0.048)^41^.

In a retrospective study from the US published in 2022 with 865 newborns who were admitted to the NICU, a positive effect of breastfeeding counselling by qualified staff on breastmilk feeding of newborns at discharge was found. A group with breastmilk feeding at discharge was compared with a group without breastmilk feeding at discharge, and the breastfeeding consultations that took place were analyzed. A higher number of breastfeeding consultations per week correlated with higher breastmilk feeding at discharge (OR=4.43; 95% CI: 1.81–10.8; p<0.0001)^37^.

Another US retrospective study published in 2019 investigated the influence of qualified breastfeeding counselling within the first 48 hours postpartum after the newborn’s admission. A significant increase in newborns fed exclusively with breastmilk on the seventh day was observed (75.6% before the introduction of breastfeeding counselling within 48 hours and 89.6% after the introduction of breastfeeding counselling within 48 hours)^42^.

In addition to breastfeeding counselling, psychological support should be offered. Mothers should be trained in breastmilk feeding and encouraged to do so because this affects the duration of breastfeeding (95% CI: 0.218–0.77; p=0.006). This was shown in a retrospective study from Greece in 2022 with 279 mothers and their newborns, who were asked about the duration of breastfeeding and the breastfeeding advice they had received^43^.

### Uninterrupted visiting hours

A prospective, Europe-wide observational study published in 2018 with 4407 participating preterm infants described a correlation between the presence of the parents and exclusive breastmilk feeding at discharge. The types of feeding (breastmilk or formula, and breastmilk supplemented with formula) were recorded regardless of the feeding method, as well as sucking at the breast. A score from 1 to 10 was created, which described parental presence based on the guidelines of the neonatal unit. This included the rules on visiting hours, the duration of visits, the opportunity for parents to be present during medical visits, and overnight accommodation on the ward. The study showed that more liberal visiting hours with a score ≥7 led to a higher likelihood of exclusive breastmilk feeding on discharge (p<0.001). This means that parents should be involved in the care of their newborns and should be allowed to stay with them day and night^44^.

Based on this narrative literature review, midwives have an important influence in all three steps of breast milk nutrition, namely in initiating lactation, strengthening the mother-child bond and providing appropriate breastfeeding advice. Therefore, direct skin-to-skin contact is recommended both after birth and in the first few days postpartum for newborns who initially require pediatric care^25-28^. Colostrum collection can be performed from 37 weeks’ gestation in all pregnant women who are at risk of hospitalization of the newborn^31,32^. When the newborn is hospitalized breast milk collection should start within the first hour postpartum^34^. It is preferable to apply the newborn to the breast. Alternatively, breast massage with hand expression should ideally be performed first, followed by electric pumping with a double pump set for 15 minutes^30,33,34,36^. Breast stimulation by the pump or the newborn should initially take place at least eight times a day. Breaks should last a maximum of 7 hours^34,35^. Direct breastfeeding should be made possible at an early stage; alternatively, non-nutritive sucking at the breast is possible^37-39^. The first breastfeeding counselling should take place within the first 48 hours postpartum and breastfeeding consultations should be held several times a week in the neonatal intensive care unit^37,40-42^. Liberal visiting times and the integration of parents in the care of newborns are measures to positively influence the success of breastfeeding^44^.

### Initiating lactation

The suckling of the newborn is responsible for the breastmilk production. If the newborn suckles more often at the breast more breastmilk will be produced^3^. If the newborn is hospitalized and not able to suckle often, appropriate help must be given to establish and maintain lactation.

In order to positively influence breastfeeding success in the long-term, the midwife can instruct the mother in manual colostrum collection from the 37th week of pregnancy onwards if it is known that the newborn needs to be admitted to the NICU after birth^31,32^. Furthermore, it is important that midwives support the mother in stimulating the breast within the first hour postpartum^34^. Ideally and normally, this is done by direct breastfeeding within the first two hours of life^37,38^. If this is not possible due to hospitalization of the newborn, a breast massage followed by hand collection of colostrum and subsequent pumping of breastmilk with a double pump set for 15 minutes several times a day, should be performed to simulate the newborns sucking patterns and hence augmenting milk production every day allowing a physiological lactation^30,33,34^. Manual collection is more effective than pumping due to the small amount of colostrum at the beginning. To prevent the mother from being overwhelmed, hand expression should be used primarily in the labor ward and pumping should be explained at the postpartum unit. Due to the lack of further studies on pumping management, the recommendations of the breastfeeding guideline should be considered. Breast stimulation by pump or the newborn should take place at least eight times a day at the beginning. To maintain milk production, when the newborn is hospitalized, pumping can occasionally be done only five times a day, but this should be an exception. Breaks should not exceed a maximum of 7 hours^35,45^. The nipples should also be viewed during the stay and the pump funnel size should be redetermined if difficulties arise. The mother should be reminded to pump during the first few nights. Breastfeeding the hospitalized newborn should be made possible at an early stage; alternatively, non-nutritive sucking at the breast can be promoted^37-39^. At home midwives can encourage the mother to continue to express breastmilk regularly, while the newborn is still in hospital^34,35,45^.

### Strengthening the mother–child bond

To support mother–child bonding early skin-to-skin contact and uninterrupted visiting hours, at best rooming-in, are recommended. If the newborn is separated by the mother due to medical conditions, every effort must be made to preserve this dyad as much as possible^3^. Therefore skin-to-skin contact should, if possible, take place within the first 30 minutes for at least 1.5 to 2 hours in the labor ward. Still, it is important that vital signs are stable, and the sick appearing newborn has been examined by a pediatrician to allow skin-to-skin contact^25-28^.

The midwife can play an important role in supporting skin-to-skin contact as she can help initiating it, as well as monitoring the infant during skin-to-skin contact and observing the newborn’s cardiorespiratory stability. When the newborn is transferred to the neonatal unit directly after birth, skin-to-skin contact should be followed up as soon as possible. A supporting midwife is very helpful in this situation. Furthermore, taking a photo for the parents to promote parent–child bonding is important^27,28^. To strengthen the parent–child bond, skin-to-skin contact can also be made at home.

The midwives and nurses on the postpartum unit should therefore facilitate regular visits by mothers to their newborns. In the best-case scenario, rooming-in of the breastfeeding mother with a sick newborn should be made possible. If this is not possible, it is very important that the parents are involved in the care of the newborn and can be with their child without limitation of visiting hours^44^. Even if the mother is immobilized, a transfer in bed to the newborn should always be possible.

### Providing appropriate breastfeeding advice

Separation of mother and child, due to the physical condition of the newborn, makes breastfeeding more difficult. A special support in hospital and at home is needed to maintain large milk supplies and to avoid frustration and failure. The breastfeeding success depends on a positive attitude, motivation and practice, and a supportive and motivating surrounding might be helpful. Lactation consultations and instructions for breastmilk pumping should take place regularly^3^.

The first breastfeeding consultation by the midwife or lactation consultant, should be carried out early, within 48 hours after birth, and breastfeeding consultations should take place several times a week in the neonatal intensive care unit^37,40-42^. Furthermore, mothers should receive psychosocial support^43^. Midwives also play an important supportive role at home in relation to successful breastfeeding^24^. Counselling on breastmilk feeding and encouraging the mother to breastfeed is particularly important. Midwives in postpartum care can provide the mother with information on breastfeeding, such as the relevance of breastmilk for the newborn and the need for regular breastmilk collection for long-term breastfeeding success^43^.

### Barriers and contraindications to breastmilk feeding

Even though MOM offers many advantages and measures to support long-term breastfeeding/breastmilk feeding have been presented, the mothers individual situation and possible obstacles or contraindications of breastmilk feeding must always be considered. If the mother is unable to breastfeed it is important to avoid blaming herself and others, and not to exaggerate the lost benefits of breastfeeding^37,45^.

On the one hand, there are personal reasons for the mother not to breastfeed, such as her own health, the circumstances of giving birth and her right of self-determination, as well as medical contraindications to breastfeeding such as a galactosemia of the newborn or maternal active herpes simplex infection at the breast, an active tuberculosis, an HIV infection depending on the viral load, or the intake of medication such as chemotherapeutics or drugs^45^.

In case of hospitalized newborns, there are further obstacles that make breastmilk feeding difficult. Parents often worry about insufficient milk supply, have difficulties with pumping and are burdened by the physical separation from the newborn during hospitalization^2^. The distance from home to the neonatal intensive care unit and transportation issues can also be an additional challenge. Furthermore, not every mother has access to a midwife or a lactation consultant who can support her with these problems^37^.

### Comprehensive algorithm for midwife-driven breastfeeding support in hospitalized newborns

**Figure 1:**
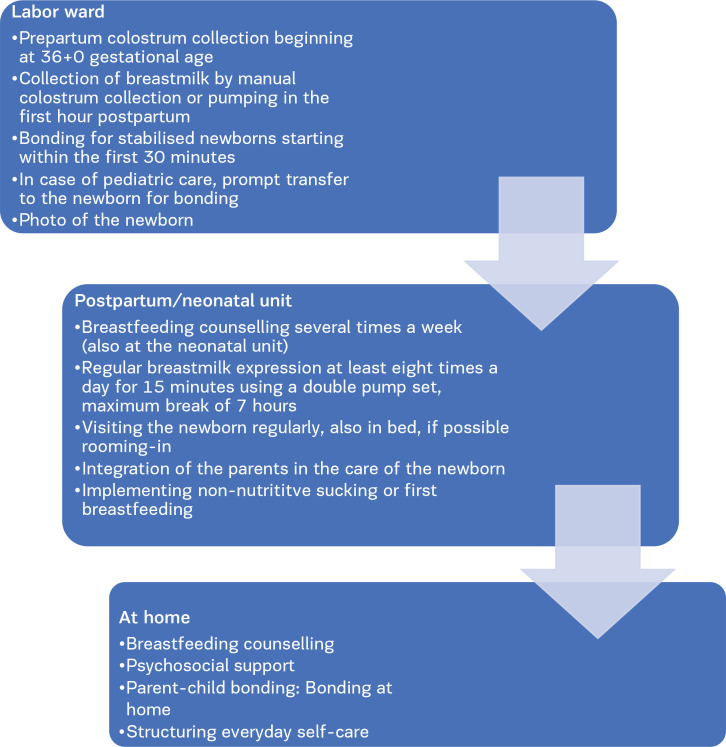
Recommendations for midwives working with women whose newborns are hospitalized

Taking into account all discussed aspects, midwives can support mothers wanting to breastfeed in multiple ways, as shown in Figure 1.

*Starting in the labor ward*

The midwife can help with pre- and post-partum colostrum collection beginning at 36+0 gestational age^30-34,37,38^. The midwife can provide support during the first skin-to-skin contact within the first 30 minutes^25-28^. In case of pediatric care, a prompt transfer to the newborn for bonding should be made possible and a photo of the newborn for mother–child bonding should be taken^25-28^.

*At the neonatal and postpartum unit*

The midwife can provide regular breastfeeding counselling several times a week, starting within the first 48 hours^37,41^. Additionally, the midwife can remind the mother to pump regularly at least 8 times a day with a maximum break of 7 hours ^34,35,45^. Rooming-in or regular visits to the newborn, even while immobilized during day and night, should be obvious and supported by the midwife^44^. First breastfeeding attempts and non-nutritive sucking should take place as soon as possible and supervised^37-39^.

*At home*

Psychosocial support and encouragement by the midwife is particularly important^24,43^. Breastfeeding counselling and pumping advice by the midwife can also be given at home^40,43^. Bonding is also possible after discharge of the newborn to support the mother–child dyad.

Supplementary file Table 2 provides a checklist of evidence-based measures that can be implemented by midwives.

### Limitations

Our study also has some limitations. Only studies from 2013 to 2023 were included with one exception^30^, due to its thematic relevance. It was difficult to find randomized controlled studies. If these were carried out, they usually had small sample sizes, which reduced their relevance. Furthermore, midwives were hardly involved. No meta-analyses meeting the inclusion criteria were found. Due to the selection of different study designs, comparability was reduced^25-44^.

Studies evaluating skin-to-skin contact were partly randomized controlled trials. None of them referred to direct postpartum skin-to-skin contact of hospitalized newborns, but rather of non-hospitalized newborns^25,26^ or skin-to-skin contact within the first days in hospitalized preterms^27,28^.

Studies evaluating breastmilk expression differed enormously in sample size (between 25 to 865 participants) and study design (prospective and retrospective studies)^29-39^. A limitation to be mentioned of a single-center study is that the questionnaire was answered retrospectively after one year, which could have distorted the results^38^. Blinding of the studies were not always possible.

The single-center study by Broom et al.^40^ did not survey the same staff 6 months before the introduction of breastfeeding advice in the neonatal intensive care unit and 6 months afterwards, which can be seen critical. Mercado et al.^41^ had large differences in the sample sizes of the two hospitals and they did not investigate the long-term effects of breastfeeding consultations on breastfeeding. In the study by Leeman et al.^42^, data were collected retrospectively before the intervention and prospectively after the intervention. Breastfeeding consultations that took place during pregnancy or in the delivery room prior to participation in the study were not considered. Also, the study did not consider the long-term success of breastfeeding. The breastfeeding or breastmilk feeding rates after discharge were not recorded^42^. Additionally, the studies on breastfeeding counselling mentioned in this article referred exclusively to postpartum breastfeeding counselling. The survey conducted by Sokou et al.^43^ did not include data on feeding patterns of newborns/infants and the initiation of breastfeeding. There is a risk that falsified information on the duration of breastfeeding was provided due to social expectations.

Overall, the studies mostly referred to premature babies. The generalizability of the results is limited as the given measures represent an ideal situation and do not consider the individual situation of the family and the NICU setting. Statistical values were not always available. Narrative reviews can be useful to summarize the current state of research but are prone to publication bias. Confounding factors like maternal health, socio-economic status or family support that might have influenced the breastfeeding success, were not always considered in the studies and therefore are not considered in this review.

## CONCLUSION

Midwives have several opportunities to positively influence the success of breastfeeding in hospitalized newborns and are a relevant contact person for the breastfeeding mother. They can support her by making skin-to-skin contact and rooming-in possible, by helping her to express breastmilk manually for colostrum collection and helping her to pump regularly for long-term breastmilk establishment. Additionally, supervision of the first breastfeeding attempts and breastfeeding counselling are important.

However, the recommendations given represent an ideal situation and cannot always be implemented for every mother and her newborn at any time. The mother’s individual situation should always be considered, as there are both medical and personal reasons why it may not be possible to feed the newborn with her own breastmilk. For reasons of medical care and stabilization of the newborn, the measures described in this review might not always be possible to be applied. Additionally, midwives are barely involved in neonatal wards, making support in breastfeeding difficult. Therefore, it is worthwhile to intensify midwifery research to involve the professional group of midwives in studies and to support an interdisciplinary approach to breastfeeding and breastmilk feeding when newborns are hospitalized. Beyond that, more randomized controlled studies on the mentioned measures are needed, as these have the highest validity and are less susceptible to bias.

## Supplementary Material



## Data Availability

The data supporting this research can be found in the Supplementary file.
