# Supplementary material for: Optimizing breastfeeding for hospitalized newborns: A narrative review of midwifery-led interventions
Source: Eur J Midwifery. 2025 Feb 21;9:10.18332/ejm/200341. doi: 10.18332/ejm/200341 (PMC11843490; doi:10.18332/ejm/200341)
Supplement: Supplementary file 1 [file EJM-9-11-s1.pdf]

Figure 1: PRISMA flowchart of literature search

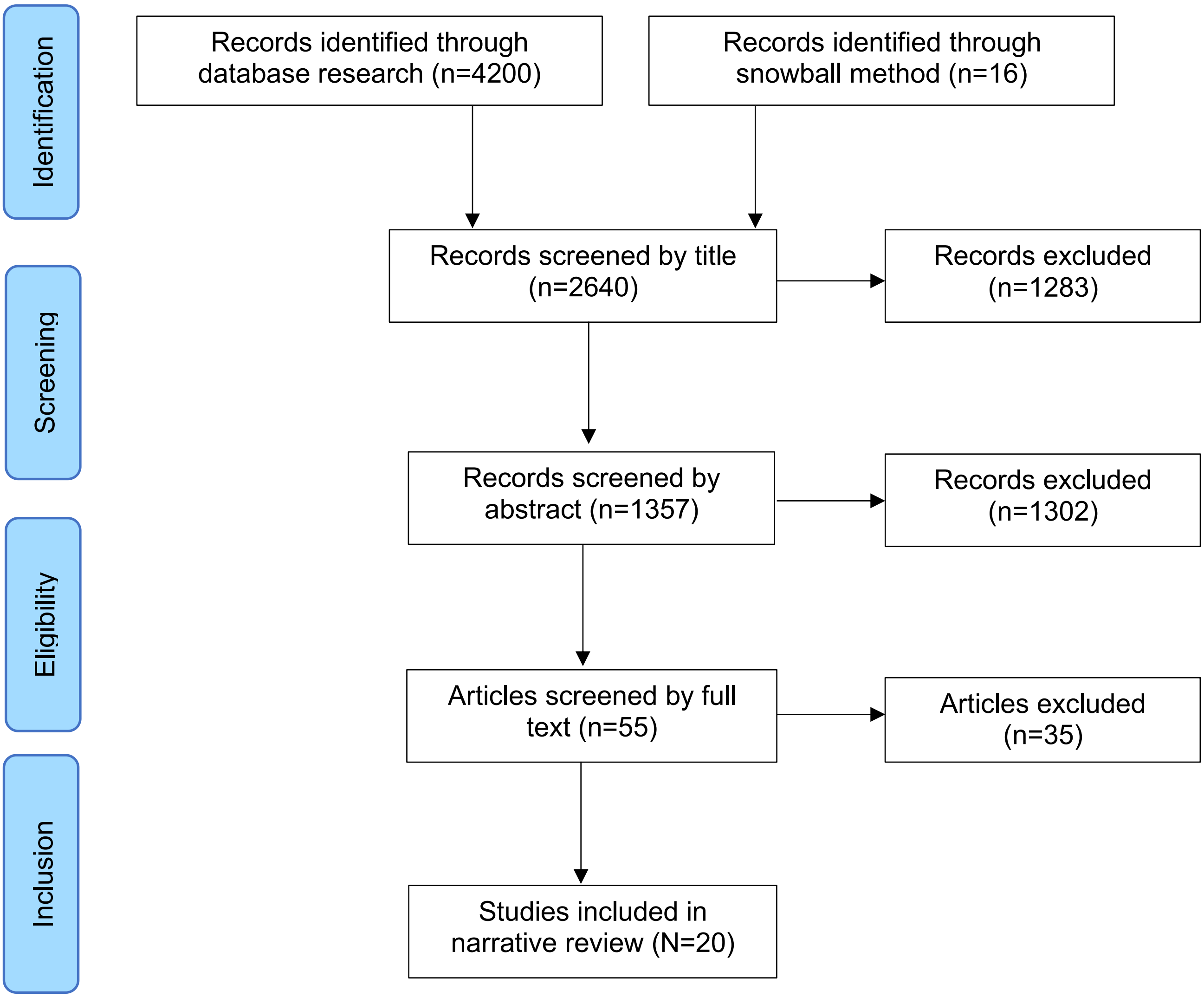

Table 1A: Search protocol of the literature search

Database	Search terms and Boolean operators	Results	Limitation to the years 2013-2023	Pre-selected records
PubMed	“Breastfeeding support” AND “Neonatal Intensive Care Unit”	784	589	13
PubMed	“Breastfeeding” AND “promotion” AND “midwife”	648	345	1
PubMed	“Breastfeeding” AND “Baby friendly hospital” AND “neonatal ward”	36	20	5
PubMed	“breast feeding success” AND “interventions” AND “newborn” OR “preterm” AND “midwife”	1276	977	4
PubMed	“breastfeeding success” AND “neonate” AND “exclusive breast feeding”	1208	484	11
CINAHL	“breastfeeding support” AND “NICU” OR “neonatal intensive care unit”	141	113	1
CINAHL	“exclusive breastfeeding” AND “NICU” OR “neonatal intensive care unit”	107	96	4
European Institute for Breastfeeding and Lactation	Snowball method			16

Table 1B: Matrix of terms relating to breastfeeding success in hospitalized newborns: Relevance of midwife care

Terms	Breastfeeding success	Measures	Hospitalized Newborns
**Related Terms**	breastfeeding support, breastfeeding success, breastfeeding, promotion, exclusive breastfeeding	interventions, midwife	newborn, preterm, neonate, Neonatal Intensive Care Unit (NICU), neonatal ward, baby-friendly-hospital

Table 2: Check list for midwives caring for mothers of hospitalized (preterm/sick) newborns

	MEASURE	DONE
**IN THE LABOUR WARD**	Prepartum colostrum collection beginning at 36+0 Gestational Age	☐
	Collection of breastmilk by manual colostrum collection (or pumping) in the first hour postpartum	☐
	Bonding for stable newborns starting within the first 30 minutes<br>In case of paediatric care: transfer of the mother to the newborn as soon as possible for bonding	☐
	photo of the newborn	☐
**POSTPARTUM UNIT/NEONATAL (INTENSIVE) CARE UNIT**	Early (within the first 48 hours) and regular (several times a week) breastfeeding counselling at the neonatal unit<br>→ Relevance of breastmilk feeding<br>→ Breastmilk expression (at least eight times a day for 15 minutes and once at night, maximum break of seven hours)	☐
	Explanation and provision of electric pump with double pump set	☐
	Rooming-in<br>Alternatively, regular visits on the neonatal ward at the newborn day and night, also in bed	☐
	Integration of the parents in the care of the newborn	☐
	Allow non-nutritive sucking at the breast or first attempts of breastfeeding	☐
**AT HOME**	Breastfeeding counselling	☐
	Parent-child bonding: Catch up on bonding	☐
	Everyday structure?<br>☐ Regular breastmilk collection<br>☐ Visits to the newborn<br>☐ Self-care of the mother	☐

© 2025 Harting K.M. et al.
